# Case Report: Anti–TNF-α therapy–associated destructive thyroiditis and unmasking of latent amyloid A amyloidosis in rheumatoid arthritis

**DOI:** 10.3389/fimmu.2026.1768736

**Published:** 2026-02-25

**Authors:** Kosuke Kumagai, Noriaki Okumura, Tomohiro Mimura, Takafumi Yayama, Mitsuhiko Kubo, Shinji Imai

**Affiliations:** 1 Department of Orthopaedic Surgery, Shiga University of Medical Science, Otsu, Japan; 2 Department of Orthopedics, Kyoto Okamoto Memorial Hospital, Kyoto, Japan; 3 Department of Spine and Joint Reconstruction, Shiga University of Medical Science, Otsu, Japan; 4 Department of Sports and Musculoskeletal Medicine, Shiga University of Medical Science, Otsu, Japan

**Keywords:** AA amyloidosis, anti-TNF therapy, destructive thyroiditis, multiple organ failure, rheumatoid arthritis

## Abstract

**Introduction:**

Tumor necrosis factor (TNF)-α inhibitors are widely used for rheumatoid arthritis (RA), but paradoxical immune reactions, including autoimmune thyroid disease, have been reported.

**Case presentation:**

We describe a 71-year-old man with a 16-year history of RA who developed destructive thyroiditis after initiation of certolizumab pegol. Despite symptom resolution, he subsequently developed acute renal failure and diarrhea. Biochemical and histological analyses revealed elevated serum amyloid A (AA) and amyloid deposition in the kidney and duodenum, confirming AA amyloidosis. We considered that the latent amyloidosis became clinically apparent following immune modulation by the anti-TNF-α biologic. Treatment with the IL-6 receptor antibody tocilizumab rapidly normalized inflammatory markers and improved both renal function and gastrointestinal symptoms.

**Conclusion:**

This case highlights that TNF-α inhibition may paradoxically unmask underlying amyloidosis and induce autoimmune thyroiditis. Clinicians should monitor thyroid and systemic amyloid markers when introducing biologic therapy for long-standing RA.

## 1 Introduction

Tumor necrosis factor (TNF)-α plays a central role in the pathogenesis of chronic inflammatory diseases, such as rheumatoid arthritis (RA). The introduction of TNF-α inhibitors has markedly improved disease control and reduced joint destruction in patients with refractory RA. However, paradoxical immune phenomena—autoimmune or inflammatory reactions that emerge despite cytokine blockade—have been increasingly reported, including psoriasis, lupus-like syndromes, vasculitis, and thyroiditis (1–4). These reactions are thought to result from complex cytokine network alterations following TNF-α inhibition (5).

Destructive thyroiditis is a self-limited inflammatory disorder characterized by follicular cell destruction and transient thyrotoxicosis. While viral infections and autoimmune mechanisms are common causes (6–8), biologic agents have also been implicated in rare cases (1, 2, 9–13). Several reports have documented thyroid dysfunction or granulomatous thyroiditis during anti-TNF therapy, suggesting that TNF-α blockade may disturb immune tolerance within the thyroid gland.

Serum amyloid A (AA) amyloidosis is another serious systemic complication of long-standing inflammatory diseases, including RA. Persistent elevation of serum AA (SAA) leads to extracellular amyloid deposition in multiple organs, most commonly the kidneys and gastrointestinal tract (3, 14–19). Although biologic therapy can reduce the risk of amyloid progression by suppressing inflammation, paradoxically, abrupt immune modulation may also alter cytokine balance and unmask subclinical amyloid deposition (18, 20–22).

To the best of our knowledge, no previous report has described the sequential occurrence of destructive thyroiditis and clinically overt AA amyloidosis following anti-TNF-α therapy. Here, we report a patient with long-standing RA who developed destructive thyroiditis and acute renal failure after treatment with certolizumab pegol (CZP). This case provides novel insight into how TNF-α inhibition can induce an immune shift that triggers both autoimmune thyroid injury and the manifestation of latent AA amyloidosis.

## 2 Case presentation

A 71-year-old man with a 16-year history of seropositive RA was referred to our department for worsening joint pain and swelling despite treatment with methotrexate (6 mg/week), salazosulfapyridine (1,000 mg/day), and low-dose prednisolone. The patient had no known family history of amyloidosis or hereditary autoinflammatory diseases. Laboratory evaluation showed elevated C-reactive protein (CRP, 7.6 mg/dL), erythrocyte sedimentation rate (58 mm/h), and matrix metalloproteinase-3 (1,650 ng/mL). Rheumatoid factor and anti-cyclic citrullinated peptide antibody levels were markedly increased (393 IU/mL and >500 U/mL, respectively). Laboratory findings before and after biologic therapy are summarized in **Table 1**.

**Table 1:** Results of blood biochemical examination before and after anti–interleukin-6 treatment.

	Onset of thyroiditis	Diagnosis of AA amyloidosis	Before tocilizumab initiation	After tocilizumab initiation	6 months after tocilizumab initiation	1 year after tocilizumab initiation
CRP (normal range is <0.30 mg/dl)	9.12	10.91	1.28	0.03	0.02	0.03
ESR 1h (<10 mm)	66.6	34.1	53.5	3.0	3.1	2.0
MMP-3 (<121.0 ng/ml)	1150	1250	1250	617	322	239
RF (<15 U/ml)	576	223	81	186	364	402
WBC (3000-8000 μl)	12000	9400	26900	7400	5500	4300
Hb (12.4-17.0 g/dl)	10.9	8.7	9.2	12.6	11.4	11.6
eGFR (>60 mL/min/1.73m^2^)	70.0	11.8	12.4	29.2	31.4	32.0
TSH (0.3-4.0µIU/1.73m^2^)	0.04	0.78	17.81	55.25	5.90	5.27
FT3 (2.5-5.0pg/ml)	4.4	0.7	0.7		1.7	1.7
FT4 (0.8-1.7ng/ml)	4.31	0.30	0.10	0.85	1.42	1.28
H-TG (<46.0ng/ml)	1000	19.3				
Amyloid (<8.0µg/ml)		461.9	21.9	8.4	3.5	2.9

CRP, C-reactive protein; ESR, erythrocyte sedimentation rate; MMP-3, matrix metalloproteinase 3; RF, rheumatoid factor; WBC, white blood cell; Hb, hemoglobin; eGFR, estimated glomerular filtration rate; TSH, thyroid stimulating hormone; FT3, free triiodothyronine; FT4, free thyroxine; H-TG, human thyroglobulin.

**Figure 1:**
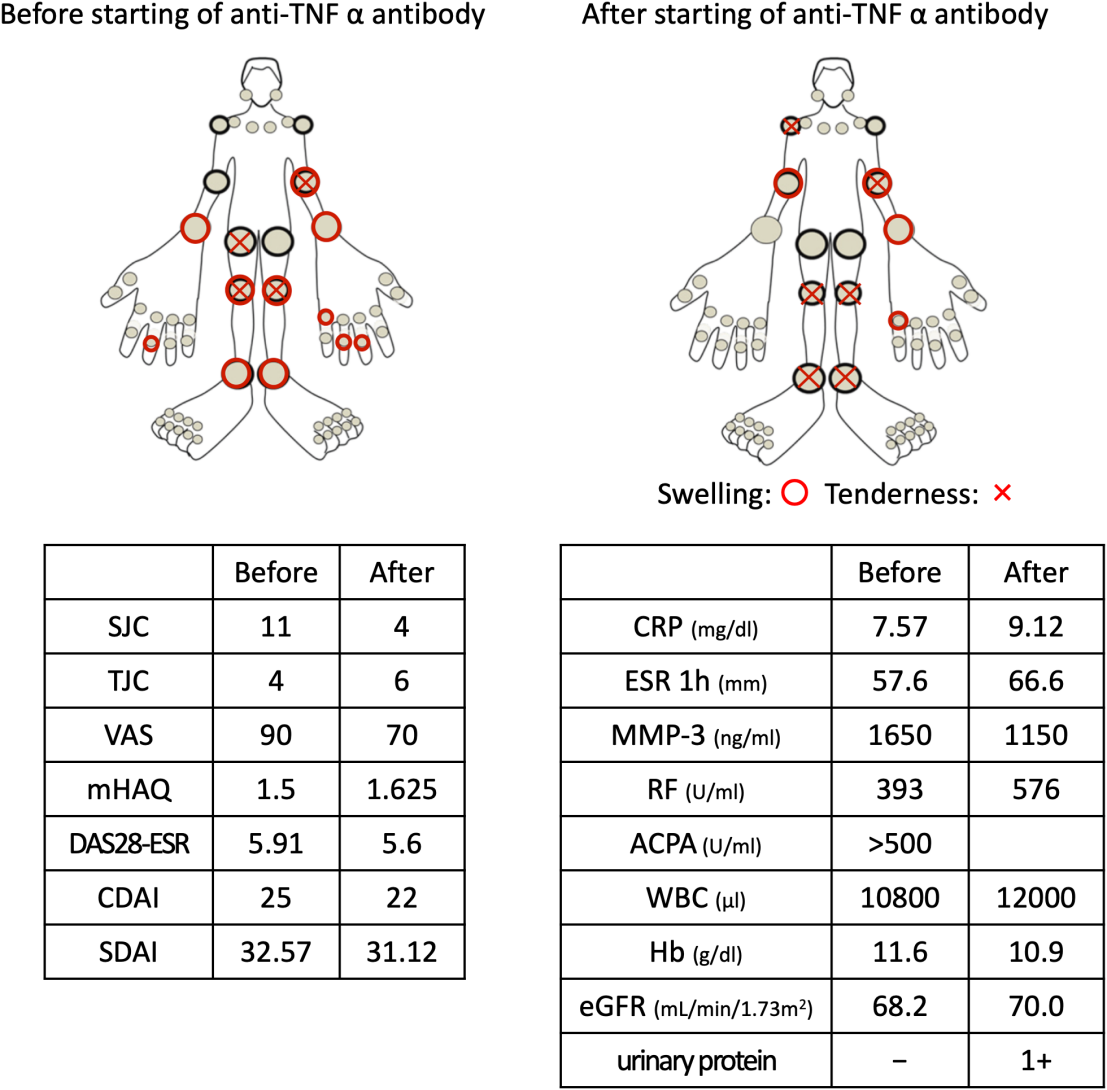
Clinical findings before and after anti-TNF-α antibody therapy. Top panels show the affected joints before and after therapy. Swelling of most joints and gripping action of the fingers improved after CZP administration. However, joint pain and blood inflammatory markers did not improve. Furthermore, rheumatoid arthritis scoring did not show any improvement. SJC, swollen joint count; TJC, tender joint count; VAS, visual analogue scale; mHAQ, modified health assessment questionnaire disability index; DAS, disease activity score 28; CDAI, clinical disease activity index; SDAI, simplified disease activity index; ACPA, anti-cyclic citrullinated peptide antibody.

Clinical findings before and after anti–TNF-α antibody therapy are summarized in **Figure 1**. Owing to inadequate disease control despite conventional synthetic DMARD therapy, certolizumab pegol (CZP), an anti–TNF-α biologic agent, was initiated. CZP was administered as a loading regimen of 400 mg subcutaneously at baseline, at week 2, and at week 4, followed by a maintenance dose of 200 mg subcutaneously at week 6.

**Figure 2:**
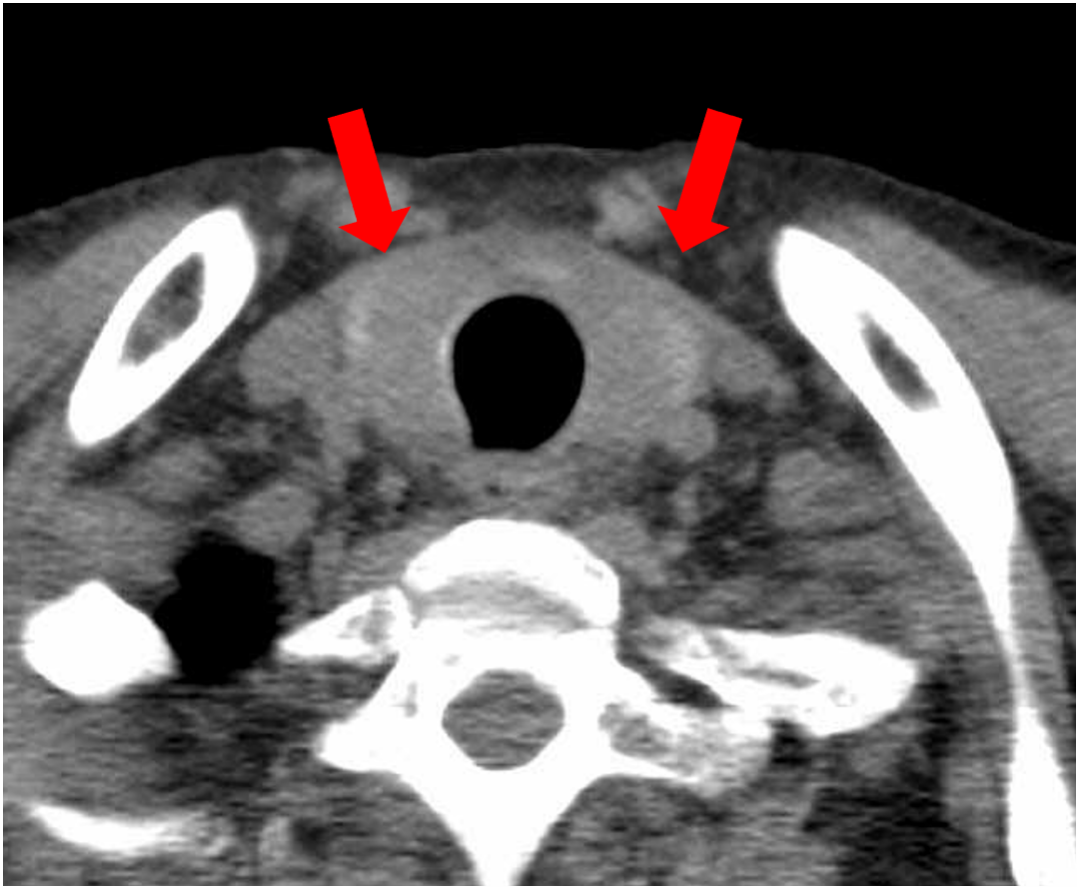
Computed tomography imaging of the thyroid on day 12 of admission. Low attenuation of the thyroid gland (red arrows) and enlarged thyroid indicated abnormal thyroid function.

After the fourth injection of CZP, the patient developed anterior neck pain, swelling, and low-grade fever. Computed tomography performed on day 12 of admission revealed diffuse enlargement of the thyroid gland with low attenuation areas (**Figure 2**). Laboratory testing indicated thyrotoxicosis (free thyroxine = 4.31 ng/dL, thyroid-stimulating hormone = 0.04 μU/mL) with markedly elevated thyroglobulin levels (1,000 ng/mL). Purulent thyroiditis was initially suspected, and cefditoren pivoxil was administered. The neck pain improved within two weeks, and thyroid function gradually normalized after discontinuation of CZP. However, systemic inflammation persisted, and two weeks later the patient developed nausea, diarrhea, and acute renal dysfunction (estimated glomerular filtration rate [eGFR] = 11.8 mL/min/1.73 m²).

**Figure 3:**
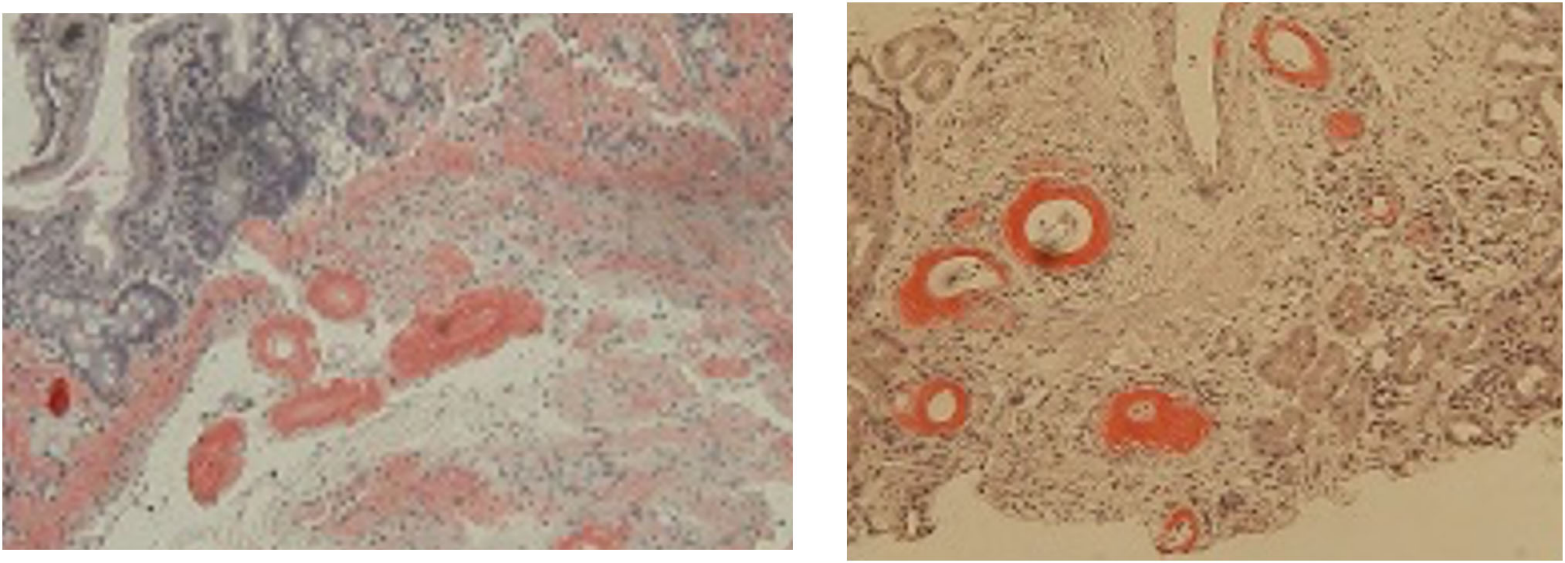
Histopathological analysis using Dyron staining of the upper gastrointestinal tract and kidney biopsies. Left panel: Upper gastrointestinal tract. From the lamina propria to the submucosa, eosinophilic and Dyron-positive substances (amyloid) were deposited around the blood vessels and in the interstitium. Right panel: Kidney. Positive Dyron staining, indicating amyloid deposition, is apparent on the blood vessel wall. Amyloid deposition was also observed in a part of the glomerulus. Lymphocytes and plasma cells, and neutrophils to a small degree, infiltrated the interstitium [100x magnification].

**Figure 4:**
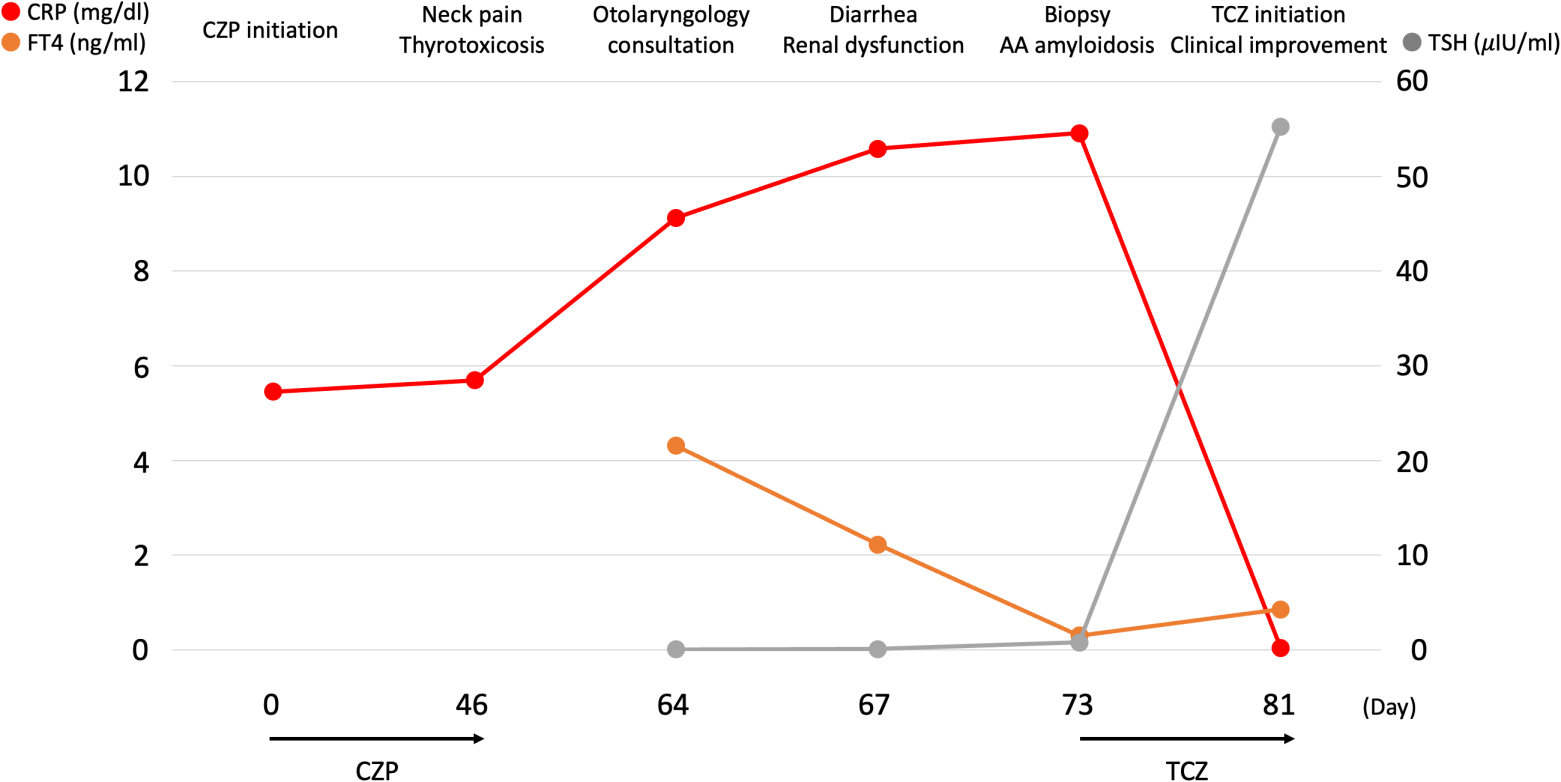
Temporal clinical course following certolizumab initiation. The x-axis represents days from the first certolizumab dose. Serum C-reactive protein (CRP) is plotted on the left y-axis, and thyroid function parameters (thyroid-stimulating hormone [TSH] and free thyroxine [FT4]) are plotted on the right y-axis. Key clinical events are indicated along the timeline, including the onset of neck pain and thyrotoxicosis, gastrointestinal symptoms with renal dysfunction, and the diagnosis of AA amyloidosis by biopsy. The timing of certolizumab pegol (CZP) initiation and subsequent switch to tocilizumab (TCZ) is annotated to illustrate the temporal relationship between biologic therapy, thyroiditis, systemic inflammation, and clinical improvement.

Despite hydration and corticosteroid therapy, renal impairment and diarrhea worsened. Serum amyloid A (SAA) was markedly elevated (461.9 μg/mL; reference <8 μg/mL). Gastrointestinal endoscopy and renal biopsy demonstrated amyloid deposition with positive Congo red staining in both the duodenal and renal interstitium, establishing the diagnosis of AA amyloidosis (**Figure 3**). The temporal clinical course is summarized in **Figure 4**. The clinical course suggested that latent amyloidosis became clinically apparent following immune modulation induced by TNF-α blockade.

Given the diagnosis of RA-associated AA amyloidosis, certolizumab was permanently discontinued and treatment with tocilizumab (TCZ, 162 mg every two weeks) was initiated. CRP levels normalized within one month, and diarrhea and renal function improved rapidly, with eGFR stabilizing at approximately 30 mL/min/1.73 m². After one year of TCZ therapy, the patient remained asymptomatic with normal thyroid function and without proteinuria.

## 3 Discussion

Although elevated serum thyroglobulin levels and a self-limited clinical course can be observed in both classic subacute thyroiditis and destructive thyroiditis, we favored destructive thyroiditis in the present case based on the overall pattern of transient thyrotoxicosis due to follicular injury rather than sustained thyroid hormone overproduction. As described in prior reviews, destructive thyroiditis is characterized by the release of preformed thyroid hormone, a transient thyrotoxic phase, and spontaneous resolution without the need for antithyroid therapy, in contrast to Graves’ disease, which is driven by ongoing hormone synthesis (23, 24).

In the present case, Graves’ disease was considered unlikely because thyroid-stimulating hormone receptor antibody (TRAb) was low (4.4%), thyroid peroxidase antibody (TPO Ab) was minimally elevated (7 IU/mL), and thyrotoxicosis resolved spontaneously after discontinuation of CZP without antithyroid therapy.

Although thyroglobulin antibody (TgAb) was modestly elevated (49 IU/mL), this finding alone was insufficient to support autoimmune hyperthyroidism, as discussed in prior reviews of thyroiditis (23, 24).

Classic subacute thyroiditis was also considered; however, the temporal association with TNF-α blockade and the clinical course following drug discontinuation suggested an immune modulation–associated destructive process. Suppurative thyroiditis was initially suspected; however, the absence of abscess formation on imaging and clinical improvement without surgical drainage argued against a bacterial etiology. Although thyroid ultrasonography and radioactive iodine uptake (RAIU) testing ware not performed, the available clinical, laboratory, and imaging findings, together with established diagnostic frameworks for thyroiditis, collectively supported the diagnosis of destructive thyroiditis (23, 24).

Systemic AA amyloidosis is a serious complication of chronic inflammatory diseases, such as RA, resulting from persistent elevation of SAA and subsequent tissue deposition (3, 14–19). Biologic agents targeting TNF-α have dramatically improved the management of RA, but paradoxical autoimmune and inflammatory reactions have also been increasingly recognized (1–4). The present case illustrates a rare phenomenon in which anti-TNF-α therapy appeared to unmask latent AA amyloidosis while simultaneously inducing destructive thyroiditis.

### 3.1 Immunological paradox of TNF-α inhibition

TNF-α regulates both pro-inflammatory and immunoregulatory pathways, and its inhibition may induce imbalance in cytokine networks, particularly through upregulation of type I interferons and IL-6 (4, 5, 25). This cytokine shift can favor autoimmunity and loss of self-tolerance. Previous reports have described subacute or granulomatous thyroiditis during anti-TNF therapy (1, 2, 9–13). Our patient developed destructive thyroiditis shortly after initiating CZP, consistent with these observations. The temporal association and absence of infectious triggers support an immune-mediated mechanism secondary to TNF-α blockade. Although subacute thyroiditis was initially considered because of neck pain and transient thyrotoxicosis, the markedly elevated thyroglobulin levels, imaging findings, and self-limited course without steroid escalation were most consistent with destructive thyroiditis.

### 3.2 A mechanistic hypothesis linking TNF-α inhibition and destructive thyroiditis

One plausible mechanistic explanation for the development of destructive thyroiditis after TNF-α inhibition involves dysregulation of innate immune responses, particularly type I interferon (IFN) signaling. TNF-α has been shown to suppress plasmacytoid dendritic cells (pDCs), a major source of type I IFNs. Inhibition of TNF-α may prolong pDC survival and enhance type I IFN production by preventing their maturation, thereby creating a pro–interferon milieu (4, 26).

Subacute and destructive thyroiditis are often triggered by viral infections and are associated with innate immune activation and interferon responses. Furthermore, IFN-based therapies have been reported to induce thyroiditis, supporting the role of type I IFN in thyroid inflammation (27). Although direct evidence is lacking in the present case, TNF-α blockade may have promoted excessive interferon signaling, predisposing the patient to destructive thyroiditis.

### 3.3 Unmasking of latent AA amyloidosis

AA amyloidosis typically develops after years of chronic inflammation and may remain subclinical until additional immune dysregulation occurs. In our patient, renal and gastrointestinal amyloid deposition became evident following the thyroiditis episode. We hypothesize that TNF-α blockade triggered abrupt cytokine rebalance, altering IL-6 and SAA dynamics and thus unmasking a previously silent amyloid deposition (18, 20, 21). A similar mechanism of “immune reconstitution” has been suggested in other biologic-induced paradoxical syndromes (4, 25), but concurrent amyloidosis and thyroiditis has not been reported.

### 3.4 Role of IL-6 inhibition in recovery

Treatment with TCZ, an IL-6 receptor antagonist, resulted in rapid normalization of inflammatory markers and recovery of renal function. IL-6 signaling drives SAA production and amyloid deposition (18, 28–30). Suppression of IL-6 by TCZ likely reduced SAA synthesis and restored immune homeostasis, consistent with prior reports showing the efficacy of IL-6 blockade in secondary amyloidosis associated with RA (28–30). Recent reports have further supported the efficacy of IL-6 inhibition in AA amyloidosis, including a case of gastrointestinal amyloidosis successfully treated with IL-6 blockade (31).

### 3.5 Clinical implications and limitations

This case emphasizes that TNF-α inhibition can paradoxically induce autoimmune thyroiditis and unmask latent systemic amyloidosis. Clinicians should carefully monitor thyroid function, renal parameters, and serum amyloid A levels during anti-TNF therapy, particularly in patients with long-standing, refractory rheumatoid arthritis or persistently elevated inflammatory markers. Early recognition of atypical immune reactions and timely switching to alternative biologic agents, such as IL-6 receptor antagonists, may help prevent irreversible organ damage.

Several limitations of this report should be acknowledged. First, this is a single case report, and therefore causal relationships between TNF-α inhibition and the observed immune phenomena cannot be definitively established. Second, subclinical amyloidosis prior to anti-TNF therapy cannot be entirely excluded, as baseline tissue evaluation was not performed. Finally, immunological biomarkers were assessed in a clinical context, and detailed mechanistic studies were beyond the scope of this report. Despite these limitations, the temporal association and clinical course provide valuable insight into paradoxical immune reactions associated with biologic therapy.

## 4 Patient perspective

The patient reported significant distress during the period of thyroiditis and renal dysfunction due to worsening physical symptoms and uncertainty regarding the diagnosis.

Following the initiation of tocilizumab therapy, he experienced marked improvement in daily activities and expressed satisfaction with the treatment outcome.

## 5 Conclusion

This case illustrates a rare but important paradoxical immune reaction associated with TNF-α inhibition in RA. The sequential development of destructive thyroiditis and overt AA amyloidosis following CZP therapy suggests that TNF-α blockade can profoundly alter cytokine homeostasis, potentially unmasking latent inflammatory complications. Our findings highlight the delicate balance between therapeutic immune suppression and unintended immune activation during biologic therapy. Monitoring thyroid function, renal parameters, and SAA levels before and during anti-TNF treatment may facilitate early recognition of such adverse events. Moreover, the rapid improvement observed after switching to an IL-6 receptor inhibitor underscores the pivotal role of IL-6 in both amyloidogenesis and paradoxical inflammation. Clinicians should remain vigilant for unexpected autoimmune or inflammatory manifestations during biologic therapy and tailor cytokine-targeted treatments according to the evolving immunological profile of each patient.

## Data Availability

The original contributions presented in the study are included in the article/supplementary material. Further inquiries can be directed to the corresponding author.
